# Extracts Rich in Nutrients as Novel Food Ingredients to Be Used in Food Supplements: A Proposal Classification

**DOI:** 10.3390/nu14153194

**Published:** 2022-08-04

**Authors:** Ricardo López-Rodríguez, Laura Domínguez, Virginia Fernández-Ruiz, Montaña Cámara

**Affiliations:** Nutrition and Food Science Department, Pharmacy Faculty, Complutense University of Madrid (UCM), Plaza Ramón y Cajal, s/n, E-28040 Madrid, Spain

**Keywords:** novel foods, novel ingredients, extracts, food supplements, risk assessment

## Abstract

Consumers’ commitment to healthy lifestyles and a varied diet has experienced rapid growth in recent decades, causing an increase in the demand of better food quality and variety. The food industry has opted for innovation and the search for new sources of food, and these trends led to the need to develop a European regulatory framework. Novel foods are under Regulation (EU) 2015/2283 (formerly Regulation (EC) No 258/97), and this concept includes all food not used in an important measure for human consumption in the EU before 15 May 1997, and which is included in any of the food categories established. Currently, there are 26 extracts authorized as novel foods or ingredients, being one of the most numerous groups. These extracts are concentrated sources of nutrients, and 23 of them can be used in food supplements. Given their heterogeneous composition and the perceptive risk assessments performed, sometimes, the authorizations are limited to certain population groups. The present work is a comprehensive review of the extracts rich in nutrients authorized as novel ingredients to be used in food supplements within the EU. A classification is proposed according to their source of origin, resulting in four main groups: extracts of plant, animal, algae, and fungal origins. A description of each extract as well as the evaluation of the potential use restriction and health benefits are also addressed.

## 1. Introduction

Consumers’ commitment to increasingly healthy lifestyles and a varied diet has experienced rapid growth in recent decades, which has led to an increase in demand in terms of the quality and variety of food and food supplements they consume. The population is increasingly aware that health encompasses something more than the mere fact of not suffering from a disease, which is why they seek to improve their well-being through an optimized diet. Thus, consumers need to acquire products such as food supplements that complement their diet in order to improve or maintain health [1,2].

The definition of food supplements, established in Directive 2002/46/EC of the European Parliament and of the Council of 10 June 2002 on the approximation of the laws of the Member States relating to food supplements, postulates them as food products whose purpose is to supplement the normal diet and consisting of concentrated sources of nutrients or other substances that have a nutritional or physiological effect, in simple or combined form, marketed in dosage form such as capsules, pastilles, tablets, pills, and other similar forms, sachets of powders, liquid ampoules, dropper bottles, and other similar forms of liquids and powders that are taken in small unit quantities [3].

Food supplements are products in the frontier of food-pharma and have a specific regulatory framework in which the legislative provisions related to extracts authorized as novel foods must be considered [1]. In this context, the food industry has opted for innovation through the application of new technological processes and the search for new sources of food and food supplements that allow it to meet these demands and thus maintain its competitiveness in a globalized market [4,5]. However, the consumers’ perception of its safety and the cost benefits associated with its consumption will be crucial in the final introduction of a novel food product on the market.

When innovations are applied to traditional foods or products, their acceptance by consumers will largely depend on the type of food and applied innovation, being well accepted those that provide relevant benefits without substantially modifying the food in question [6]. The lack of positive consumer perception regarding the potential benefits of these products can lead most consumers to question the need and usefulness of this novel product, and may even have the opposite effect, increasing the perception of risk [7]. That is why it is common to carry out consumer studies (during the stages of identifying a new product, its development, or the tests before its commercialization) as a step prior to launching a new product on the market [8].

These innovation trends in the market caused the need to develop an adequate regulatory framework in the European Union, thus giving rise to the so-called novel foods. The concept of novel food, initially established by Regulation (EC) No. 258/97, concerning novel foods and novel food ingredients, and currently in force according to Regulation (EU) 2015/2283, includes any food that has not been used in an important measure for human consumption in the European Union before 15 May 1997, and that is included in any of the food categories established in the aforementioned Regulation [9].

The authorization procedure for extracts as novel foods has followed different paths depending on whether the authorization took place [9]. Until 1 January 2018 (date of entry into force of the current Regulation (EU) 2015/2283), the applicant must present a request to the Member State in which the novel food was placed on the market for the first time. The Member State in question issued an initial assessment report with the conclusions about the safety of the novel food. If no substantiated objection was raised, the novel food could be placed on the market. If additional evaluations were required, the novel food had to be the subject of a further assessment by the European Food Safety Authority (EFSA) [10]. After completing the evaluation requested, the EFSA sent it to the European Commission who, in turn, prepared a draft Decision. This draft was sent to the Standing Committee on the Food Chain and Animal Health (SCFCAH), where the Decision was definitively adopted by authorizing or denying the placing on the market of the novel food in question.

Parallel to the general authorization procedure for novel foods, Article 5 of Regulation (EC) No. 258/97 established a simplified procedure called notification, applicable to those foods and food ingredients that were substantially equivalent to existing foods or food ingredients regarding their composition, nutritional value, metabolism, intended use, and content of undesirable substances, in accordance with the scientific data available, recognized by general form and/or opinions issued by one of the competent bodies. In this procedure, the applicant had to notify the placing of the product on the market to the European Commission. This notification had to be accompanied by a favorable scientific opinion issued by a competent body of one of the Member States.

After the entry into the force of the current Regulation (EU) 2015/2283, all valid applications for the evaluation of novel foods are sent by the European Commission to the EFSA, which has a maximum period of 9 months to proceed with its evaluation and issue an opinion on the safety of the novel food. Once its evaluation is complete, the EFSA publishes its opinion and transmits it to the Commission, the Member States, and the applicant. Within 7 months from the date of publication of the EFSA’s opinion, the Commission will submit to the Standing Committee on Plants, Animals, Food and Feed (SCPAFF) a draft implementing act, in accordance with the procedure laid down in Regulation (EU) No. 182/2011, which authorizes the marketing of the novel food in the European Union and updates the so-called Union list of novel foods established in Regulation (EU) 2017/2470 [11,12]. Novel foods are under the food legislation approved in the European Union so that they must meet the labeling provisions established by Regulation (EU) No. 1169/2011 [9]. Additional labeling requirements regarding a particular characteristic or property of the novel food in question (nutritional composition and value, intended use, restriction of use in specific population groups, etc.) are included in Commission Implementing Regulation (EU) 2017/2470, which establishes the Union list of novel foods in accordance with Regulation (EU) 2015/2283 of the European Parliament and of the Council on novel foods. The objective of the present work is to study the authorized extracts as novel foods or food ingredients for its use in food supplements in the European Union since 1997. In order to group the different types of extracts authorized, a classification of these extracts is proposed by the authors of the present work. A description of each extract as well as the evaluation of the potential use restriction and health benefits are also addressed in this study.

## 2. Materials and Methods

A search for scientific opinions, regulations, and bibliographies has been carried out using official databases such as Web of Science and Science Direct. A literature review of the current and available scientific evidence about the potential health benefits attributed to these extracts rich in nutrients was performed in scientific databases and resources such as Pubmed and Google Scholar, using the name of each extract plus “health benefits” as selected keywords. Scientific studies published in the English language and during the last 11-year period (2011–2022) were considered in the present review.

The authorization procedure for extracts as novel foods for its use in food supplements has taken place through different pathways, established both in Regulation (EC) No. 258/97 and in Regulation (EU) 2015/2283. The analysis of the different authorization decisions and regulations, and the reports of initial and complementary evaluations of the different extracts, allows us to know their main characteristics as sources of nutrients and the main aspects related to their safety evaluations, as well as establishes a classification of the same.

This study focused only on those novel foods of which the word ‘extract’ is included in the title of the EU decision as a classification and selection purpose. The authors are aware that other novel foods already approved include an extraction process; however, those are not approved as extracts, and are thus not included in this proposed classification.

## 3. Results and Discussion

To date, a total of 26 extracts has been approved as novel foods and novel food ingredients in the European Union. The great majority of these extracts (23/26) has been authorized to be used in food supplements (FS). Out of these 23 extracts, 20 were authorized under the former Regulation (EC) No. 258/97 on novel foods. The authorization of red cranberry powder extract and three-root extract (*Cynanchum wilfordii* Hemsley, *Phlomis umbrosa* Turcz., and *Angelica gigas* Nakai) was requested through the former Regulation; however, their evaluation had not been completed when the current Regulation (EU) 2015/2283 entered into force on 1 January 2018, therefore the authorization process of these two extracts took place under the current Regulation, after a new assessment of their safety by the EFSA. Finally, the remaining extract (extract of *Panax notoginseng* and *Astragalus membranaceus*) was evaluated and authorized following the current procedure established by Regulation (EU) 2015/2283 [9].

The great majority of the extracts (15/23) were placed on the market through the notification procedure (substantial equivalence), while another five extracts were authorized through decisions. The three remaining extracts have been authorized through implementing regulations, in accordance with the procedure established in Regulation (EU) 2015/2283 [9].

**Figure 1:**
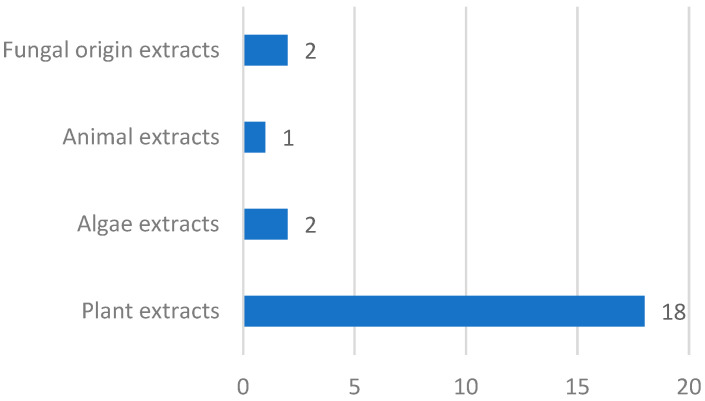
Distribution of 23 extracts authorized to be used in food supplements according with Novel Food Regulation.

In order to group the different types of extracts authorized for use in food supplements, a classification of these extracts is proposed by the authors of the present work according to the extract origin, distinguishing for these four main groups: extracts of plant origin (18/23), algae extracts (2/23), extracts of animal origin (1/23), and extracts of fungal origin (2/23) (Figure 1). Extracts of plant origin can be further classified into four subgroups: seed extracts; extracts of leaves, fruits, and roots; plant cell culture extracts; and other extracts of plant origin. In each group, the date of the authorization of each extract has been considered as a criterion for ordering them.

In the case of extracts authorized as novel foods for its use in food supplements, the group of extracts of plant origin is the largest and, therefore, the most likely to be studied in the scientific literature. Another factor to take into account is that many food supplements have a complex composition, resulting from the mixture of several substances, therefore it may not be easy to identify the origin of an adverse effect. The review of the scientific literature has made it possible to identify some examples of possible adverse effects associated with this type of substances.

### 3.1. Extracts of Plant Origin

The use of substances of plant origin is common in the elaboration of food supplements, and particularly those based on botanical products and plant extracts have also experienced rapid growth [13]. This fast rise has led to numerous scientific studies’ investigation of the potential beneficial or adverse effects associated with its consumption [14,15].

**Figure 2:**
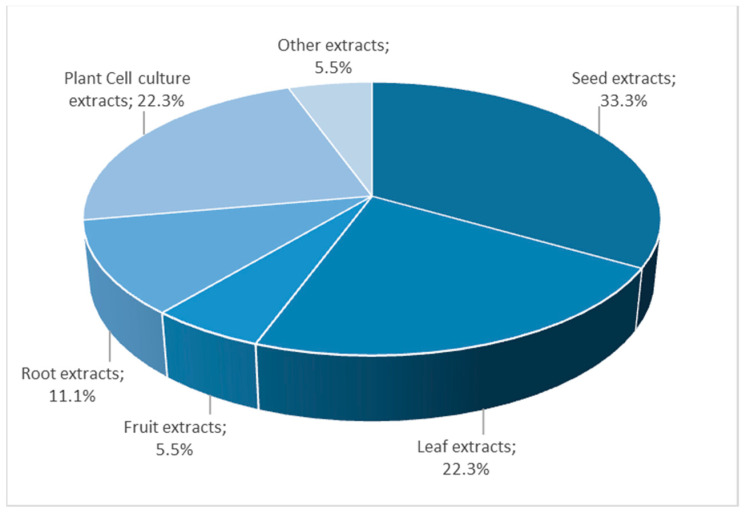
Distribution of extracts of plant origin considered in this study.

A total of 18 extracts are included in this group, being the largest group of extracts authorized to date to be used in food supplements. Within this category, the subgroups of extracts of seeds and the extracts of leaves, fruits, and roots would be the most numerous, including six and seven extracts, respectively, followed by the subgroup of plant cell culture extracts with four extracts (Figure 2).

#### 3.1.1. Seed Extracts

This group includes six extracts: sunflower oil extract, extract of defatted cocoa powder, low fat cocoa extract, fermented soybean extract, fermented black bean extract, and spermidine-rich wheat germ extract (*Triticum aestivum*) (Table 1).

The first extract marketed within this group was the sunflower oil extract, placed on the market for the first time in 2009 through the notification procedure, when substantial equivalence was established for its use in food supplements with the corn germ oil extract with a high content of unsaponifiable material, which had previously been authorized as a novel food by Decision 2006/723/EC. Substantial equivalence was established on a weighted basis of 1 g of sunflower oil extract with 2 g of corn oil extract [16]. This sunflower oil extract is characterized by its high content of oleic and linoleic acids (20 and 70%, respectively) in addition to phytosterols (5.5%) and tocopherols (1.1%) [12]. According to the results of the European Project “Using rapeseed and sunflower meal as novel ingredients” (November 2017–December 2021), side products obtained from sunflower oil can be used as potential novel ingredients in the food industry. For instance, de-oiled sunflower kernels can be considered as a promising new protein source with some food applications, such as a meat analogue [22].

With regard to cocoa extracts, both were marketed for the first time through the notification procedure, having established substantial equivalences with a defatted cocoa powder, in the case of extract of defatted cocoa powder (for its use in food supplements and other foods) [18], and with a natural cocoa powder in the case of low fat cocoa extract for its use in food supplements [19]. Defatted extract and low fat cocoa extract are rich in polyphenols (minimum 55% gallic acid equivalent) and flavonols (minimum 300 mg/g), respectively. These high contents gave rise to additional specific labeling requirements in order to inform consumers that they should not consume neither more than 600 mg of polyphenols per day, which is equivalent to 1.1 g of the extract of defatted cocoa powder, nor more than 600 mg of cocoa flavanols per day, in the case of low fat cocoa extract [12]. Oddoye et al. (2013) reported that some by-products of cocoa beans such as cocoa pulp juice, also known as “sweatings”, are used as ingredients in several food products. Cocoa pulp juice stands out for its natural content in sugars (glucose, fructose, and sucrose) and minerals (calcium, magnesium, and potassium, among others) [23]. This by-product can be added to refreshing drinks, either alone or in combination with other fruit juices. It can be used for making jam as well. Other food applications, such as the production of alcoholic beverages (gin, brandy, wine) and vinegar, can be obtained with the fermentation of the sugars naturally present in cocoa pulp juice [23].

On the other hand, two of the other extracts included in this group have their source in soybeans. The fermented black bean extract, authorized by Decision 2011/497/EU for its use in food supplements [20], is rich in protein (≥55%) and contains an alpha-glucosidase inhibitor. This extract was subject to a complementary assessment by the EFSA (2011) as the initial evaluation report received several comments and objections related, among others, to the toxicological information provided. Even though the toxicological and clinical studies provided limited evidence on the safety of the extract in question, the EFSA considered that it did not pose a concern. The target population of food supplements containing this extract were adults who wanted to inhibit the digestion of carbohydrates in order to control their weight [24]. In this sense, it should be highlighted that the authorization of a novel food is based on its safety, so that any health claims must be authorized in accordance with the procedure established for this purpose in Regulation (EC) No. 1924/2006 on nutrition and health claims on foods [25]. Regarding the potential health benefits, Kim et al. (2011) carried out in vitro, in vivo, and ex vivo clinical trials with black soybean extract to investigate its effect on platelet activation, an important risk factor in cardiovascular diseases [26]. The results suggested that black soybean extract could be able to attenuate thrombosis through the inhibition of collagen-induced platelet activation, opening the doors for this extract’s use as a novel food supplement in the management of cardiovascular disorders and for the improvement of blood circulation [26].

Regarding the fermented soybean extract, it contains the enzyme nattokinase (20,000–28,000 UF/g) extracted from natto, which results from the fermentation of non-genetically modified soybeans (*Glycine max* L.) to which a selected strain of *Bacillus subtilis* var. Natto is added. This parenterally administered enzyme has fibrinolytic activity in vitro and thrombolytic activity in vivo in animals, as revealed by EFSA (2016a) in its complementary evaluation [27]. Due to this activity, it was established as a specific requirement for additional labeling that food supplements containing this extract contain a warning stating that people taking medicines must consume the product exclusively under medical supervision [28]. In addition to this specific requirement, the two soy extracts must be labeled in accordance with Annex II of Regulation (EU) No. 1169/2011, given that the risk of allergic reaction to these soy extracts is similar to that of other soy products [9]. Park et al. (2013) performed an in vitro study with soybean extract and demonstrated a potential modulation of retinoic acid-related gene expression of skin and photo-protective effects in human keratinocytes [29]. These preliminary results could be a good starting point to further investigate the use of soybean extract in food supplements intended to protect skin from the damage caused by UVB irradiation [29].

The spermidine-rich wheat germ extract is the last extract of this group authorized (December 2017) by means of substantial equivalence with the wheat germ of *Triticum aestivum* (common wheat) for its use in food supplements [21]. This extract is characterized by its contents of spermidine (0.8–2.4 mg/g) and spermine (0.4–1.2 mg/g). Scientific studies carried out on supplementation with spermidine, an autophagy-inducing agent, have shown a protective effect against neurodegeneration and cognitive impairment in animal models [30]. Nutritional and functional composition of wheat germ, an important by-product of the flour milling industry, is characterized by its content of protein (26–35%), sugars (17%), lipids (10–15%), minerals (4%), fiber (1.5–4.5%), as well as significant amounts of certain bioactive compounds such as tocopherols (300–740 mg/kg dry matter), phytosterols (24–50 mg/kg), and carotenoids (4–38 mg/kg) [31]. According to several in vitro and in vivo clinical trials, the fermented wheat germ extract, a food supplement commercialized under the name of Avemar^®^, has shown potential health benefits in rheumatoid arthritis, cardiac remodeling, and metabolic symptoms [32,33].

The fermented soybean extract, resulting from the fermentation of non-genetically modified soybean (*Glycine max* L.), can be considered as an example of seed extract for which potential adverse effects have been identified in the scientific literature. Di Lorenzo et al. (2014) identified 95 scientific publications regarding adverse effects associated with *Glycine max* (L.), mainly related to its allergenic potential or its isoflavone content (used to reduce menopausal symptoms) [34,35,36].

#### 3.1.2. Extracts of Leaves, Fruits, and Roots

This group includes four leaf extracts: alfalfa leaf extract (*Medicago sativa*), Aloe macroclada Baker leaf extract, aqueous extracts of dried leaves of *Ilex guayusa* and *Epigallocatechin gallate* as purified extract of green tea leaves (*Camellia sinensis*); one fruit extract: powdered cranberry extract; and two root extracts: three-root extract (*Cynanchum wilfordii* Hemsley, *Phlomis umbrosa* Turcz., and *Angelica gigas* Nakai), and *Panax notoginseng* and *Astragalus membranaceus* extract (Table 2).

##### Extracts of Leaves

Alfalfa leaf extract is the only one of this group authorized by Commission Decision. It is an extract rich in proteins (45–60%) whose use is only authorized in food supplements [37]. During its initial assessment carried out by the French competent authorities, various safety issues were highlighted, leading the initial assessment report to conclude that a further evaluation was necessary. It is an extract that has used since 1992 in third-world countries outside the European Union to combat malnutrition without adverse effects having been detected. The complementary evaluation carried out by the EFSA (2009) focused on the presence of phytoestrogens (Coumestrol and isoflavones) and L-canavanine, ruling out the presence of adverse effects based on studies in humans and animals [44]. In this sense, maximum contents were set for these substances in the specifications. Another key aspect to assess was the allergenic potential, concluding that the existence of a cross-reaction in subjects allergic to peanuts could not be ruled out. The final authorization decision (Decision 2009/826/EC) did not establish any specific provision regarding this issue [37]; however, Regulation (EU) No. 1169/2011, on food information provided to the consumer, is applicable and establishes as mandatory the mentioning of any ingredient or technological aid that appears in its annex II or derives from a substance or product that appears in that annex that causes allergies or intolerances and is used in the manufacture or processing of a food and is still present in the finished product, even if in a modified form [9]. The nutritional and functional composition of alfalfa leaf has been widely studied in the scientific literature. Alfalfa leaf contains interesting amounts of fiber, vitamins, minerals, chlorophylls, carotenoids, and phytoestrogens. Moreover, it has been reported that alfalfa leaf can be considered a good source of phenolic compounds (quercetin, naringenin, kaempferol, medicarpin, luteolin, myricetin, apigenin, etc.) and bioactive compounds with an important antioxidant activity and potential antimicrobial, anti-inflammatory, and immunomodulatory properties. On the other hand, and in accordance with some preclinical studies, alfalfa leaf extract enriched with vitamin C could strengthen and enhance the immune system. It has also been suggested that the use of this extract as a food supplement could be beneficial in some disorders of the digestive tract as well as in malnutrition and ischemic disease. However, further studies are needed to confirm these potential health effects in human organisms [45].

Regarding the other three extracts, it should be noted that they were placed on the market for the first time through the notification procedure for their use in food supplements. *Epigallocatechin gallate* extract, as a purified extract (≥90%) of green tea (*Camellia sinensis*) leaves, was commercialized upon the establishment of substantial equivalence to other green tea extracts with a history of safe use prior to 1997 [38]. *Epigallocatechin gallate* is the main component of the polyphenolic fraction of green tea, responsible for most of the therapeutic effects attributed to its consumption, highlighting its antioxidant and anti-adipogenic potential [46,47]. A systematic review carried out by Momose et al. (2016) resulted in 17 human trials showing a potential capacity of *Epigallocatechin gallate* extract in decreasing LDL levels after 4–14 weeks of supplementation [48]. Recently, Chatree et al. (2020) performed an in vitro study with human adipocytes and revealed that the administration of *Epigallocatechin gallate* extract reduced triglycerides concentrations as well as systolic and diastolic blood pressure after 8 weeks of supplementation [49].

In the case of the *Aloe macroclada* Baker leaf extract, substantial equivalence was established with an *Aloe vera* L. leaf extract with a history of safe use in food supplements prior to 1997 [39]. It stands out for its content in dietary fibers (28.6%), polysaccharides (9.5%), and glucose (8.9%) [12]. According to the scientific literature, Aloe gel obtained from the leaf has potentially showed antiviral and immunological properties as well as hypoglycemic activity in human clinical trials. For that reason, Aloe gel has been used in some food supplements for the management of different diseases or disorders such as acquired immune deficiency syndrome and diabetes [50].

The systematic review carried out by Di Lorenzo et al. (2014), regarding the possible adverse effects of *Camellia sinensis* (L.) Kuntze [34], identified 34 publications, 29 of which were considered as sufficiently documented to assess their causality. Among the side effects described, acute hepatoxicity is underlined, including cases with clinical effects described as a slight increase in serum aminotransferases levels or even hepatitis [34].

These potential adverse effects were associated with different degrees of causality, with food supplements based on green tea extracts, including hydroalcoholic extracts, and with aqueous extracts of green tea consumed either as a tea or in capsules, being the gallic esters of catechins, and in particular of Epigallocatechin-3-gallate, the compounds most frequently identified as responsible in cases of hepatotoxicity [40]. However, it is necessary to highlight that most of the cases described were classified as “certain/probable” or “possible” when considering the potential contribution of other factors such as age, concomitant pathological conditions, the presence of other ingredients, or even adulteration or contamination [34].

Aqueous extracts of dried leaves of *Ilex guayusa* were considered to be substantially equivalent to aqueous extracts of *Ilex paraguariensis* for its use in food supplements and infusions. This extract stands out for its caffeine content (19.8–57.7 mg/100 g)—one of the main study parameters when establishing equivalence, as differences related to caffeine content were found between both extracts. Natural variability based on the location, the type of crop, the collection, and the extraction process could be the reason for such differences [51]. Among the main potential beneficial effects of guayusa, it is important to highlight its stimulant and antioxidant properties. A systematic review performed by Radice et al. (2016) with an extract of dried leaves of *Ilex guayusa* [52] reported a reduction in hyperglycemia in animal models. Further scientific studies are needed to elucidate the potential use of this extract in nutraceutical formulations.

##### Extracts of Fruits

To date, this subgroup is made up of a single authorized extract: cranberry extract powder, characterized by its high content of phenols (>46.2%, expressed as gallic acid equivalents) and proanthocyanidins (55.0–60.0% or 15.0–18.0%, depending on the analytical method used). The initial application included its use in various types of beverages and by several population groups, including children. In this sense, during their initial evaluation, emphasis was placed on the possible risks existing for children aged 1–3 years old due to the potential excessive consumption of polyphenols through the novel food and other sources present in the diet of these children [41]. This issue, along with other objections, led to a further evaluation by the EFSA (2017) and the modification of the application excluding infants, young children, and adolescents from its use, focusing the requested uses only on adults. The EFSA concluded that the use of this extract in the proposed conditions was safe considering the estimation of the intake together with the results of clinical studies in humans without adverse effects [53].

However, the European Commission continued to show concern about the risk that infants, young children, and adolescents could consume these drinks with the extract in question. For this reason, the alternative of authorizing cranberry extract for its use in food supplements intended for the adult population was proposed. This authorization is currently in force according to Regulation (EU) 2018/1631 [41].

It is well known that cranberry extracts are commonly used in food supplements to alleviate some symptoms of acute and uncomplicated urinary tract infections. A systematic review carried out by Gbinigie et al. (2020) [54], found some human studies in which cranberry extract capsules were associated with a within-group improvement in urinary symptoms.

A comprehensive review carried out by Kowalska and Olejnik (2016), [55], comprising 7 human studies and 10 animal studies suggested that cranberry extract could be used as an effective complement in individuals with metabolic complications as it could be able to ameliorate insulin resistance, improve plasma lipid profile, and reduce diet-induced weight gain and visceral obesity as well as different markers of oxidative stress. Peixoto et al. (2018) demonstrated that cranberry extract [56], could enhance the metabolic profile and decrease the oxidative damage and steatosis in rats with a high-fat diet. These studies suggest that the administration of cranberry extracts through food supplements could be helpful in managing obesity-related disorders along with the pharmacological treatment.

##### Extracts of Roots

This group is made up of two extracts: the extract formed by three herbal roots (*Cynanchum wilfordii* Hemsley, *Phlomis umbrosa* Turcz., and *Angelica gigas* Nakai), and the extract from *Panax notoginseng* and *Astragalus membranaceus*.

The extract of three herbal roots (*Cynanchum wilfordii* Hemsley, *Phlomis umbrosa* Turcz., and *Angelica gigas* Nakai) is characterized by its content of some compounds such as phenols (13.0–40.0 mg/g), coumarins (13.0–40.0 mg/g), and iridoids (13.0–40.0 mg/g). The proportion of this mixture of roots is the following: 32.5% (p/p) of *Cynanchum wilfordii*, 32.5% (p/p) of *Phlomis umbrosa*, and 35.0% (p/p) of *Angelica gigas* [12]. The initial application, which established its use in food supplements aimed at postmenopausal women, was the subject of a complementary evaluation by the EFSA (2016) wherein the safety of this extract was not established for the requested maximum intake level (514 mg/day), as this exceeds the level of intake considered safe. However, the EFSA concluded that the extract was safe for adults if it was added to food supplements at a maximum daily dose (175 mg/day), which was significantly lower than that initially requested, and which corresponded to the safe intake level. Likewise, it was considered that the risk of allergic reaction to *Angelica gigas* Nakai did not differ from celery, since both plants belong to the same botanical family (*Apiaceae*) [57]. After providing additional information by the applicant, the EFSA carried out a new evaluation, where it reaffirmed the conclusions of its first report [58].

The final authorization of this extract for its use in food supplements intended for the adult population [42], established as a specific labeling requirement that food supplements containing the extract of the mixture of the three herbal roots will include, next to the list of ingredients, the indication that it should not be consumed by people allergic to celery, in accordance with the provisions of Regulation (EU) No. 1169/2011 [9].

An in vivo study performed by Oh et al. (2018) [59], suggested a potential improvement of stress-induced depression in mice after supplementation with the extract of three herbal roots (*Cynanchum wilfordii* Hemsley, *Phlomis umbrosa* Turcz., and *Angelica gigas* Nakai). These authors indicated that those potential health effects could be attributed to the antagonistic activity on the 5-HT6 receptor. Due to its possible antidepressant effects, this extract is starting to attract the attention of both food and pharmaceutical industries; however, human studies are needed to confirm these preliminary findings.

The extract from *Panax notoginseng* and *Astragalus membranaceus* is the last authorized extract (in 2020) as a novel food [60]. It is a mixture of two extracts: an ethanol extract from the roots of *Astragalus membranaceus* (Fisch.), Bunge, and a hot water extract from the roots of *Panax notoginseng* (Burkill) F.H. Chen, fundamentally characterized by a content of carbohydrates (≥90%), proteins (≤4.5%), and saponins (1.5–5%) [43].

This extract is authorized by Regulation (EU) 2020/1821 for its use in food supplements (maximum content 35 mg/day), as defined in Directive 2002/46/EC, for the general adult population, except food supplements for pregnant women, after having been the subject of a risk assessment by the EFSA (2020), in which, among other issues, the extensive history of the use of the two plants used was revealed, especially in traditional Chinese medicine. Regarding its toxicity, a safe intake of 0.5 mg/kg body weight/day (corresponding to a maximum daily intake of 35 mg) was established based on a no adverse effect level (NOAEL) of 100 mg/day/kg body weight/day, derived from a subchronic toxicity study, and applying a safety factor of 200. Furthermore, the presented studies ruled out any concern regarding genotoxicity. Another aspect evaluated by the EFSA was its potential allergenicity, given the presence of proteins (≤4.5%) in its composition. Considering the extensive history of the use of the two plants used in the production of the extract, it was concluded that the risk of possible allergic reactions, although unknown, was expected to be low in the case of the general population [60].

As a specific additional labeling requirement, Regulation (EU) 2020/1821 establishes that the labeling of food supplements containing *Panax notoginseng* and *Astragalus membranaceus* extract will include a statement highlighting that these food supplements should not be consumed by individuals under the age of 18 years old or pregnant women [43].

According to Zhou et al. (2012) [61], *Panax notoginseng* and *Astragalus membranaceus* are considered Chinese medicinal plants. *Panax notoginseng* contains ginsenosides, bioactive compounds with potential health effects such as immunological and anti-fatigue functions. *Astragalus membranaceus* is known as a tonic to strengthen the immune system and, in combination with ginseng, it is used in Chinese medicine to manage certain ailments.

#### 3.1.3. Plant Cell Culture Extracts

A total of four extracts are included in this group: *Ajuga reptans* extract, *Echinacea angustifolia* extract, dried extract of *Lippia citriodora*, and *Echinacea purpurea* extract. All of them were placed on the market for the first time through the notification procedure for their use in food supplements, with the *Echinacea purpurea* extract being the last one commercialized (2017) (Table 3).

Regarding the substantial equivalences established with foods with histories of safe uses, the *Ajuga reptans* extract was substantially equivalent to the extracts of the flowering aerial parts of *Ajuga reptans* obtained through traditional cultivation [62]; *Echinacea angustifolia* extract to the root extract of *Echinacea angustifolia* obtained in ethanol-water titrated to 4% echinacoside [63]; *Lippia citriodora* extract to a similar extract of leaves of *Lippia citriodora* obtained by traditional cultures [64]; and *Echinacea purpurea* extract to a similar extract of the flower of the chapter of *Echinacea purpurea* [65].

Unlike other authorized extracts, no specifications about their composition or the content of undesirable substances have been established in the Union List [12] for the four abovementioned cell culture extracts. Only specifications about the description of these cell culture extracts have been set. No specific maximum amounts have been established for its use in food supplements; however, reference is made to quantities consistent with a normal use of the extracts with which the substantial equivalences were established. 

Di Lorenzo et al. (2014) identified 20 publications related to possible adverse effects of *Echinacea purpurea* (L.); however, these side effects are mainly associated with aqueous and hydroalcoholic extracts of roots and herbs, while the one authorized as a novel food is a dry extract and was authorized in 2017 [34].

Reported effects include allergenicity, mainly due to IgE-mediated hypersensitivity as a consequence of the immunostimulatory properties of *Echinacea purpurea* [34,66,67] and acute hepatotoxicity [68,69].

Esposito et al. (2020) performed a study on *Ajuga reptans* extract. The results showed that this extract could decrease the reactive oxygen species levels in cancer cell lines, opening the doors for its use as an active ingredient for nutraceutical or pharmaceutical purposes [70]. Toso and Melandri (2011) investigated the possible benefits attributed to *Echinacea angustifolia* extract from cell cultures in intensive human sport. A significant reduction in lipoperoxides levels (oxidative stress marker) in 20 humans under high physical training was demonstrated after a daily supplementation during 4 weeks with an *Echinacea angustifolia* plant cell culture extract that contained of 2.5 mg echinacoside [71]. In an in vitro study, Ghasempour et al. (2016) suggested the antifungal activity of an ethanolic extract of *Lippia citriodora*, which could be a good starting point to further investigate in vivo its efficacy and properties [72]. Motamedi et al. (2018) confirmed that *Echinacea purpurea* extract has beneficial effects on sperm characteristics in mice. According to the results of this in vivo study, the extract in question significantly increased the sperm count as well as its motility and mobility [73]. In addition, Banica et al. (2020) indicated that food supplements and extracts of *Echinacea purpurea* have antiviral, antibacterial, or antioxidant activities [74]. Among the main active substances present in *Echinacea purpurea*, polyphenols mainly derived from caffeic acid should be highlighted.

#### 3.1.4. Other Extracts of Plant Origin

A taxifolin-rich extract from the wood of Dahurian Larch (*Larix gmelinii* (Rupr.) Rupr), which had no history of safe use in the European Union, is included in this group. This extract stands out for its high content of the flavonoid taxifolin (≥90.0% of dry weight), an antioxidant used in a wide range of food products, including food supplements [75,76].

In 2010, the initial application of this extract included several uses (alcoholic beverages, chocolate products, yoghurts, and food supplements) which were initially evaluated by the competent authorities of the United Kingdom [75]. Subsequently, the abovementioned uses were subject to a positive complementary evaluation by the EFSA, basing the risk characterization on the calculation of the margin of exposure (MOE) of the combined intake [77]. However, its use was only authorized in food supplements intended for the general population, excluding infants, young children, children, and adolescents under 14 years of age [78]. Then, the Commission requested a new evaluation from the EFSA about the rest of the uses and levels of use whose authorization had not finally been granted. After informing the applicant, the applicant requested a further extension of the use and conditions of use in dairy products intended for the general population, as well as a change in the chemical name of taxifolin. Finally, after the evaluation carried out by the EFSA, based on the estimation of the exposure for the new uses and calculation of the MOE, Regulation (EU) 2018/461 authorized the rest of the uses initially requested and the extension to the dairy products [79,80].

Wang et al. (2011) suggested that extract rich in taxifolin and other flavonoids from the wood sawdust of *Larix gmelinii* showed a remarkable antioxidant activity measured by the DPPH and BHT assays [81]. These antioxidant properties are important since scientific evidence has demonstrated that oxidative stress caused by reactive oxygen species (ROS) is considered one important risk factor for the appearance of different chronic diseases and disorders. However, more studies are crucial to clarify the potential health effects of this extract as well as its use in the food and pharmaceutical industries.

### 3.2. Algae Extracts

This group comprises two fucoidan extracts from the algae *Fucus vesiculosus* and *Undaria pinnatifida*, both placed on the market for the first time in 2017 by the notification procedure for their use in foods and food supplements after a joint evaluation of their safety [82] (Table 4).

Both fucoidan extracts from the algae *Fucus vesiculosus* and *Undaria pinnatifida* are allowed to be commercialized in two types of extracts depending on the concentration of fucoidan. Thus, in the case of *Undaria pinnatifida*, the concentration of this compound varies between 75–95% in one of the extracts and 50–55% in the other, while, in the case of *Fucus vesiculosus*, the concentrations of fucoidan vary between 75–95% and 60–65% [12].

Fucoidan is a sulfated polysaccharide characterized by its high content of L-fucose and sulfate, as well as other minor components such as xylose, galactose, mannose, and glucuronic acid. As examples of its biological activity, some authors include the antioxidant, anti-inflammatory, antiviral, or antitumor activities as well as its effect on osteoblastic differentiation [83].

Bae et al. (2020) [84], evaluated the potential effect of fucoidan extracted from *Fucus vesiculosus* on ovarian cancer. The preliminary results revealed that this extract was able to inhibit in vitro the development of human ovarian cancer through different mechanisms, suggesting the potential use of this fucoidan extract in the pharmaceutical industry [84]. According to the review published by Zhao et al. (2018) [85], fucoidan extracted from *Undaria pinnatifida* could be considered an interesting source for nutraceuticals and functional foods given its antioxidant and antiviral properties.

### 3.3. Extracts of Animal Origin

A protein extract from pig kidneys is included in this group. This extract of animal origin was placed on the EU market for the first time in 2012 through the notification procedure for its use in food supplements and foods for special medical uses [17,86].

It is an extract with a natural content of the enzyme diamine oxidase (DAO) that was initially formulated as enteric-coated capsules to target the active sites of digestion and with its use limited to three capsules daily (0.9 mg/day DAO). This authorized use was extended by Regulation (EU) 2020/973 [87], without an evaluation by the EFSA, to include enteric-coated tablets, so that the maximum quantity currently authorized in food supplements is “3 capsules or 3 tablets/day; equivalent to 12.6 mg of pig kidney extract per day. Diamine oxidase (DAO) content: 0.9 mg/day (3 capsules or 3 tablets with a DAO content of 0.3 mg/capsule or 0.3 mg/tablet)” [12].

To the authors knowledge, there is no scientific evidence about the potential health effects of pig kidney extract. The literature review in different scientific databases carried out by the authors of the present work did not find any study assessing these effects.

### 3.4. Extracts of Fungal Origin

To date, only two extracts have been authorized within this group: mushroom chitosan extract (*Agaricus bisporus*; *Aspergillus niger*) and shiitake mushroom mycelium extract (*Lentinula edodes*) (Table 5).

On one hand, the chitosan extract from fungi (*Agaricus bisporus*; *Aspergillus niger*) stands out for having been the first extract authorized as a novel food in 2008 for its use in food supplements, and which was placed on the EU market for the first time through the notification procedure as substantial equivalence with a crustacean chitosan extract has been established [88]. This extract is characterized by its chitosan content (≥85%) which, in turn, mainly contains poly D-glucosamine. A maximum amount of use in food supplements has not been established, but it will be in line with the normal use in these food supplements of chitosan from crustaceans with which substantial equivalence was established [12].

Among the uses of chitosan, the formulation of food supplements and its use in the treatment of hypercholesterolemia or the prevention of cardiovascular risks should be highlighted [90,91].

On the other hand, the mycelial extract from Shiitake mushroom (*Lentinula edodes*) is a sterile aqueous extract obtained from the mycelium of *Lentinula edodes* cultivated in submerged fermentation. This extract was authorized by Decision 2011/73/EU [89] for its use as a novel food ingredient in various foods and in food supplements, standing out for its lentinan content (0.8–1.2 mg/mL), a modifier of the biological response with immunostimulatory properties [92]. The competent authorities of the United Kingdom evaluated the safety of this β-glucan and its estimation of exposure [93], which were in turn one of the aspects that the EFSA underlined in its complementary evaluation along with its potential allergenicity. Its risk was not considered higher than that derived from the consumption of the *Lentinula edodes* mushroom and other sources of β-glucan [94].

Shiitake mushroom has traditionally and commonly been used as a food ingredient in the Asian culture, specifically in China and Japan, whereas its use in American and European cuisines is currently increasing. Scientific evidence about the potential medicinal value of shiitake mushrooms (*Lentinula edodes*) is scarce. It has been suggested that shiitake mushrooms have immune-modulating, antitumor, and antiviral properties; however, further randomized, double-blind, and controlled clinical trials need to be performed to clarify these benefits [95]. Regarding the mushroom chitosan extract (*Agaricus bisporus*; *Aspergillus niger*), no specific scientific studies were found in the literature review performed.

## 4. Conclusions

After an intensive study of the extracts rich in nutrients and bioactive compounds authorized as novel food ingredients to be used in food supplements within the European Union, the results indicate that the extracts are one of the largest groups within the novel foods or novel food ingredients authorized, with currently 26 extracts authorized. These extracts are characterized as concentrated sources of nutrients. Out of the 26 authorized extracts, 23 of them include food supplements among their authorized uses. Given its heterogeneous composition, and considering the perceptive risk assessments carried out, the authorizations are limited, in some cases, to the use of the extract in food supplements to certain population groups. A classification of these extracts according to their source of origin was proposed by authors in the present work, and this results in four main groups: (a) extracts of plant origin (classified into four subgroups: seed extracts; extracts of leaves, fruits, and roots; plant cell culture extracts; and other extracts of plant origin), (b) algae extracts, (c) extracts of animal origin, and (d) extracts of fungal origin. This proposed classification could be considered a useful approach to obtain an organized description of each extract and a useful tool in the evaluation of their potential use restriction.

The use of plant-based substances is common in the production of food supplements, which have also experienced a rapid growth, particularly those based on botanical products and plant extracts. This fast rise has meant that the potential beneficial or adverse effects associated with its consumption have become subject of study in numerous scientific studies.

In the case of extracts authorized as novel foods for its use in food supplements, the group of extracts of plant origin is the largest and, therefore, the most likely group to be studied in the scientific literature, although a greater part of them have been authorized in the last 4 years. Another factor to consider is that many food supplements have a complex composition, resulting from the mixture of several substances, therefore it may not be easy to identify the origin of an adverse effect. The review of the scientific literature performed in the present work has made it possible to identify some examples of adverse effects associated with this type of substances.

The potential health effects attributed to the extracts rich in nutrients have been identified in the scientific literature. Among the most interesting benefits suggested, the possible use of some of these extracts in cardiovascular diseases (fermented black bean extract, Alfalfa leaf extract), rheumatoid arthritis (fermented wheat germ extract), metabolism disorders (fermented wheat germ extract, cranberry extract), disorders of the digestive tract (Alfalfa leaf extract), urinary tract infections (cranberry extract), ovarian cancer (fucoidan extracted from *Fucus vesiculosus*), stress-induced depression (extract of three herbal roots: *Cynanchum wilfordii* Hemsley, *Phlomis umbrosa* Turcz., and *Angelica gigas* Nakai), hyperpigmentation (Aloe leaf extract), skin protection (soybean extract), hyperglycemia (extract of dried leaves of *Ilex guayusa*), maintenance of normal blood pressure and cholesterol levels (*Epigallocatechin gallate* extract), strengthening of the immune system (Alfalfa leaf extract, extracts from *Panax notoginseng* and *Astragalus membranaceus*), improvement of physical performance (*Echinacea angustifolia* extract) and sperm characteristics (*Echinacea purpurea* extract), antifungal activity (ethanolic extract of *Lippia citriodora*), and antiviral properties (fucoidan extracted from *Undaria pinnatifida*) could be highlighted. Regarding the pig kidney extract and mushroom chitosan extract (*Agaricus bisporus*; *Aspergillus niger*), no scientific evidence about their possible health effects was found in the literature review carried out by the authors of the present work.

In conclusion, these extracts could be used as novel ingredients in functional products such as functional foods, food supplements, and even nutraceuticals to complement the daily diet and contribute to the maintenance of an adequate health status. However, further studies in humans are necessary to clearly demonstrate and confirm these preliminary results, as most of the studies found in the scientific literature were carried out in vitro.

## Figures and Tables

**Table 1:** Seed extracts authorized as novel foods for its use in food supplements.

Extract	Maximum Levels in Food Supplements	Initial Evaluation	Authorization
Sunflower oil extract	1.1 g/day	France	Substantial equivalence [16,17]
Extract of defatted cocoa powder	1.0 g/day	Ireland	Substantial equivalence [17,18]
Low fat cocoa extract	1.2 g/day	Ireland	Substantial equivalence [17,19]
Fermented soybean extract	100 mg/day	Belgium	Decision (EU) 2017/115 [20]
Fermented black bean extract	4.5 g/day	United Kingdom	Decision (EU) 2011/497/EU [20]
Spermidine-rich wheat germ extract	6 mg/day spermidine	Austria	Substantial equivalence [17,21]

**Table 2:** Leaf, fruit, and root extracts authorized as novel foods for its use in food supplements.

Extract		Maximum Levels in Food Supplements	Initial Evaluation	Authorization
Leaf extracts	Lucerne leaf extract from *Medicago sativa* (Alfalfa leaf extract)	10 g/day	France	Decision 2009/826/EC [37]
	*Epigallocatechin gallate* as purified extract from green tea leaves (*Camellia sinensis*)	150 mg in one portion	Ireland	Substantial equivalence [17,38]
	*Aloe macroclada* Baker leaf extract	In line with normal use in food supplements of the similar gel derived from *Aloe vera* (L.) Burm.	Ireland	Substantial equivalence [17,39]
	Aqueous extracts of dried leaves of *Ilex guayusa*	In line with normal use in herbal infusions and food supplements of a similar aqueous extract of dried leaves of *Ilex paraguariensis*.	Ireland	Substantial equivalence [17,40]
Fruit extracts	Cranberry extract powder	350 mg/day.	France	Regulation (EU) 2018/1631 [41]
Root extracts	Extract of three herbal roots (*Cynanchum wilfordii* Hemsley, *Phlomis umbrosa* Turcz. and *Angelica gigas* Nakai)	175 mg/day (for adult population).	Ireland	Regulation (EU) 2018/469 [42]
	Extract from *Panax notoginseng* and *Astragalus membranaceus*	35 mg/day (for adult population).	EFSA	Regulation (EU) 2020/1821 [43]

**Table 3:** Cell culture extracts authorized as novel foods for their use in food supplements.

Extract	Maximum Levels in Food Supplements	Initial Evaluation	Authorization
*Ajuga reptans* extract from cell cultures	In line with normal use in food supplements of a similar extract of the flowering aerial parts of *Ajuga reptans*	Italy	Substantial equivalence [17,62]
*Echinacea angustifolia* extract from cell cultures	In line with normal use in food supplements of a similar extract from the root of *Echinacea angustifolia*	Italy	Substantial equivalence [17,63]
Dried extract of *Lippia citriodora* from cell cultures	In line with normal use in food supplements of a similar extract from the leaves of *Lippia citriodora*	Italy	Substantial equivalence [17,64]
*Echinacea purpurea* extract from cell cultures	In line with normal use in food supplements of a similar extract from florets within the flower head of *Echinacea purpurea*	Italy	Substantial equivalence [17,65]

**Table 4:** Algae extracts authorized as novel foods for its use in food supplements.

Extract	Maximum Levels in Food Supplements	Initial Evaluation	Authorization
Fucoidan extract from the seaweed *Fucus vesiculosus*	250 mg/day	Belgium	Substantial equivalence [17,82]
Fucoidan extract from the seaweed *Undaria pinnatifida*	250 mg/day	Belgium	Substantial equivalence [17,82]

**Table 5:** Extracts of fungal origin authorized as novel foods for its use in food supplements.

Extract	Maximum Levels in Food Supplements	Initial Evaluation	Authorization
Chitosan extract from fungi (*Agaricus bisporus*; *Aspergillus niger*)	In line with normal use in food supplements of chitosan from crustaceans	Belgium	Substantial equivalence [17,88]
Mycelial extract from Shiitake mushroom (*Lentinula edodes*)	2.5 mL/day	United Kingdom	Decision 2011/73/EU [89]
